# On the structure of grain/interphase boundaries and interfaces

**DOI:** 10.3762/bjnano.5.172

**Published:** 2014-09-22

**Authors:** K Anantha Padmanabhan, Herbert Gleiter

**Affiliations:** 1 School of Engineering Sciences & Technology and Centre for Nanotechnology, University of Hyderabad, Prof. C. R. Rao Road, Hyderabad 500 046, India; 2 Karlsruhe Institute of Technology (North Campus), Institute of Nanotechnology, Helmholtz Platz, 76344 Eggenstein, Germany

**Keywords:** geometrical approach, grain/interphase boundaries, interfaces, representative volume, structural/basic unit model

## Abstract

Grain/interphase boundaries/interfaces of varying misorientations, free volume fractions, curvatures and irregularities are present in materials, both 3D and 2D, regardless of whether these materials are crystalline or amorphous/glassy. Therefore, a question arises about the central idea on which a general description of grain/interphase boundaries/interfaces can and should be based. It is suggested that a generalized model of a structural/basic unit (crystalline, non-crystalline or of any scale), which depends on the interatomic (including electronic) interactions, the spatial distribution of the atoms and electrons, the number of atoms and free volume fraction present in the structural/basic unit and the experimental conditions should serve the purpose. As the development of a quantitative model, which reflects the effects of all these variables is difficult, slightly defective material boundaries are often modeled by treating the entire boundary as planar and by using the concepts of crystallography. For highly disordered boundaries, a description in terms of a representative volume, made up of a non-crystalline basic unit or a combination of such units, which depend on interatomic (including electronic) interactions and forces, is advocated. The size, shape, free volume fraction and number of atoms in the representative volume could differ with material composition and experimental conditions. In the latter approach, it is assumed that all processes connected to a problem on hand is contained within this representative volume. The unresolved issues are identified.

## Introduction

What is reported below is a full account of an invited talk delivered at "INT Physics Days", held at the Institute of Nanotechnology, Karlsruhe Institute of Nanotechnology (North Campus), Germany, in November 2013. Successful attempts have been made to understand some of the properties of materials by using geometrical notions based on the symmetry of atomic arrangements. There are also properties that critically depend on defects and their distribution in materials. For explaining at least some of the latter class of properties, ideas based on geometry/crystallography are only of limited use [1]. At the other extreme is the notion of fractals, which has also found use in describing microstructures [2–3].

The advent of metallic glasses [4] and nano-glasses [5–6] has made it clear that a purely geometrical/crystallographic description of the structure of grain/interphase boundaries and interfaces (found in nano-glasses, where the glassy regions on either side are non-crystalline or when an electrode–electrolyte system is subjected to a voltage) is not likely to be tenable as a general concept. It seems fair to say that even before the discovery of metallic glasses, geometrical consideration was only a necessary, but not sufficient, condition. For example, not all properties associated with grain boundaries (GBs) in all face-centered cubic (fcc) metals, e.g., the presence or absence of an orientation-dependence of the grain boundary energy, are identical, although the geometry of their atomic structure is the same. In this communication we examine if there is a concept that will allow a unified description of grain/interphase boundaries and interfaces in materials of different kinds. In our view the concept of a *“structural unit”* [7]*, after sufficient generalization*, could serve this purpose.

We suggest that, at the level of atomistics, the free volume fraction and the number of atoms present in an ensemble should also be treated as variables and that even the short-range order that develops need not always lead to crystallinity. When the ensemble is crystalline, we have referred to it as “structural unit”. The non-crystalline variant is termed as “basic unit”. It is further suggested that when these structural and basic units are mixed in different proportions, one could obtain three different types of grain/interphase boundaries/interfaces, viz., (a) crystalline boundaries that have long-range order, (b) general/random high-angle boundaries and metallic/bulk metallic melt-quenched glasses, which do not have long-range order, (c) vapor-quenched and compacted nano-glasses, which, compared with (b), contain in addition basic units of much greater free volume fraction, which give rise to new properties. The description and the behavior of these three types of boundaries/interfaces are different. As a consequence of our research interests, in the distant past H. Gleiter started from (a) downward. In contrast, K. A. Padmanabhan started from (b). The underlying thread of connectivity among the different ideas is the focus of this paper.

## Review

### Structural complexities: A qualitative description

The central idea in our approach is that the magnitude and nature (metallic, ionic, covalent, molecular, etc.) of interatomic (including electronic) interactions, number of atoms and free volume fraction present in a system and the experimental conditions are responsible for the development of the structural/basic units. The final atomic configurations within these structural/basic units will be decided by the minimization of total free energy and, depending on experimental conditions, maximization of entropy to the extent possible. The structures that are formed need not always be crystalline, neither in the bulk nor in the grain/interphase boundaries and interfaces. Each boundary/interface will comprise many “structural/basic units”, all of which are not necessarily identical. They could also be of rather complex configurations, i.e., the structural/basic units that constitute a grain/interphase boundary/interface could be a collection of different ensembles of atoms whose number, size and shape are governed by the well-known free energy equation

[1]
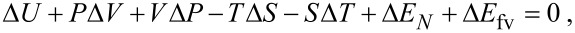


where *U* is the internal energy, *P* the pressure, *V* the volume, *T* the absolute temperature, *S* the entropy, *E_N_* the energy component that changes with the number of atoms (*N*) present in the ensemble mainly because of topological reasons and *E*_fv_ is the energy associated with the free volume (fv) present in the aggregate. (In the traditional form of Equation 1, the energy contributions from *N* and fv are absorbed into *U*. But, as the present discussion is concerned with the formation of the structural/basic units at an atomistic level, in our opinion, it is essential to treat *N* and fv also as independent variables for unequivocal inference.) “Δ” denotes the infinitesimal changes in the above quantities due to a transformation/reaction. That is, the size, number and shape of the structural/basic units will be decided by the total number of atoms present in the system, their mutual affinity or tendency to form compounds (if more than one species is present), the initial temperature and the energy possessed by the atoms at the beginning of the transformation, the final temperature, change in pressure and volume due to a transformation, excess free volume and the rate of cooling/quenching. To the best of our knowledge, a truly random distribution of atoms is not found either in pure elements or when more than one species is present in a material, i.e., short-range order always exists. This is due to the presence of interatomic (including electronic) attractive and repulsive forces and the drive to decrease the free energy of the system. Equation 1 applies to a cluster of atoms. When it gets embedded inside a solid matrix, the energy associated with the insertion of the atom cluster into the solid matrix also has to be included. Eshelby [8] has explained how this can be done in a quantitative manner by approximating the shape of the basic unit to an oblate spheroid. This will lead to an average value. For irregular ensembles, a quantitative procedure is yet to be developed.

Sometimes ensembles of a small number of atoms, which are unique arrangements of lowest free energy, are formed [9]. These configurations depend on the number of atoms and the interatomic/chemical potential (including electronic interactions and those due to their spatial distribution). It is not clear if such configurations are possible in non-metals and if it is not possible, the reason for the same is not known. Such a structure may have an elastic modulus greater than that of the bulk. Free energy increases even if the number of atoms is increased or decreased by one [10]. When the number of atoms is larger than *N*, for large *N*, the following states of matter are found: crystalline materials (conventional and nano-crystalline), melt-cooled glasses and “ultra-stable” nano-glasses with distinctly different properties. It is interesting that to this day nano-glasses have been produced by using only compositions out of which melt-quenched metallic glasses have been formed in the past. In all these types of materials, the grain boundaries/interfaces have a well-defined structure (not necessarily crystalline) with respect to density, elastic properties and Mössbauer spectrum. At present, there is no priori method of estimating the value of *N*.

**Figure 1:**
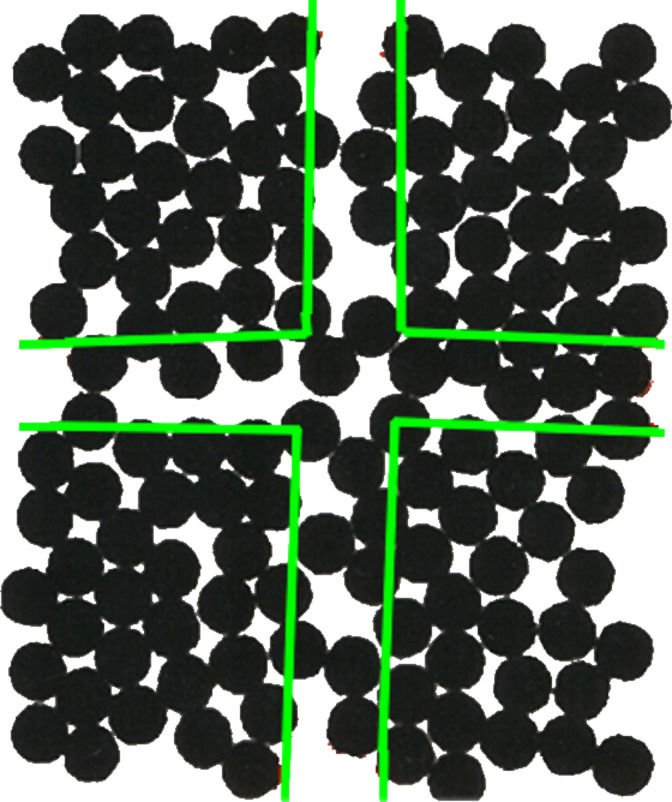
Schematic representation of the interface in a nano-glass [5].

Nano-glasses (Figure 1) are produced from polymeric [11–13] as well as metallic materials [5,14–15]. Detailed structural studies on polymeric nano-glasses are sparse. The processing route seems to be important: Metallic nano-glasses are made by using physical vapor deposition or sputtering, while polymeric nano-glasses are made by using vapor deposition or laser-assisted vapor deposition. In polymeric nano-glasses there is also some evidence that the substrate temperature has a significant effect on properties. From structural studies and the measurements of interfacial properties (e.g., density, Young’s modulus) of metallic nano-glasses it is known that the atomic arrangements and electronic structures are different from what is found in the interface of relaxed glassy structures as well as the grain boundaries of crystalline materials [5,14]. When these differences are ignored and the interatomic forces present in melt-cooled glasses, as used in MD simulations, are used in the calculations, the time of stability of nano-glasses is predicted to be about 10^−6^ s or less. In reality, however, the nano-glasses are stable for months, even longer [5]. In our opinion, this huge difference is because in MD simulations mostly empirical potentials intended for crystalline materials are used and such potentials are not relevant to situations far from the crystalline state. This point of view is also in agreement with the observation that the interfaces in certain nano-glasses (e.g., Fe_90_Sc_10_) are ferromagnetic, whereas the corresponding melt-quenched Fe_90_Sc_10_ glass is paramagnetic at the same temperatures.

Moreover, radial distribution measurements have revealed that nano-glasses have a longer and special short-range order (SRO) compared with melt-quenched glasses. This is qualitatively assigned to the greater energy release during vapor-quenching compared to melt-quenching. (The ratio of heat of vaporization to heat of fusion is 25.33 for iron, 19.05 for scandium, 23.55 for palladium, 49.03 for lithium, 37.36 for beryllium, 19.38 for phosphorus, 10.16 for boron and so on. Similar values for polymers are not available.) In this line of argument, this enormous energy release raises the local temperature and enables the diffusive ordering of atoms over a longer range in nano-glasses than in melt-quenched alloys. One may even hazard a guess that for compositions prone to the formation of metallic glasses the greater the ration of heat of vaporization to the heat of fusion, the greater will be the tendency to form a nano-glass. In terms of Equation 1, in nano-glasses the terms *T*Δ*S* and *S*Δ*T* are very significant and thus the formation of nano-glasses is accompanied by high entropy changes. These structures seem to depend on the experimental conditions, the total number of atoms in the ensemble and free volume fraction. To date, generally accepted methods for determining the atomic (including electronic) interactions and the corresponding potentials for situations where symmetry-based arguments fail are not available.

In polymer nano-glasses the glass transition temperature is significantly raised. In contrast, in conventionally quenched polymer glasses, a change in the cooling rate by several orders of magnitude has very little effect on the glass transition temperature. In one experiment involving a metallic nano-glass the glass transition temperature was raised by 25 °C [9]. The density was decreased by about 40% (in one experiment the density actually increased [12–13]) but the Young’s modulus was similar to or higher than that of the crystalline and the denser, conventional glassy counterparts [9–10,14]. This could indicate a change in the nature of electronic interactions, e.g., from metallic to covalent bonding. The heat capacity was 20% lower, but the enthalpy and the kinetic stability were higher compared with the melt-quenched glass variant. The rule of thumb that thermal stability is proportional to density is violated. (With a qualification “as long as the nature of the bonding between atoms does not change”, the above rule of thumb could still be useful.) Interfaces in nano-glasses are often wider than in crystalline materials. It stands to reason that when a material is quenched from the temperature of vaporization, the quenched-in free volume will be significantly more than when the atoms are quenched in from the melt-quenching temperatures. This can explain the lower density of the nano-glasses.

In a recent study [16], it has been shown that lattice expansion or amorphization makes EuTiO_3_ ferromagnetic, although the stable phase of crystalline EuTiO_3_ is antiferromagnetic. Ferromagnetism increases with an increase in the lattice volume of EuTiO_3_. Amorphization also has a similar effect on ferromagnetism. This observation has been explained in terms of competition between ferromagnetic and antiferromagnetic interactions among the Eu^2+^ ions. Similar ideas could also be relevant in understanding the development of ferromagnetism in Fe_90_Sc_10_ nano-glasses, which in the melt-spun and crystalline states is paramagnetic [15].

**Figure 2:**
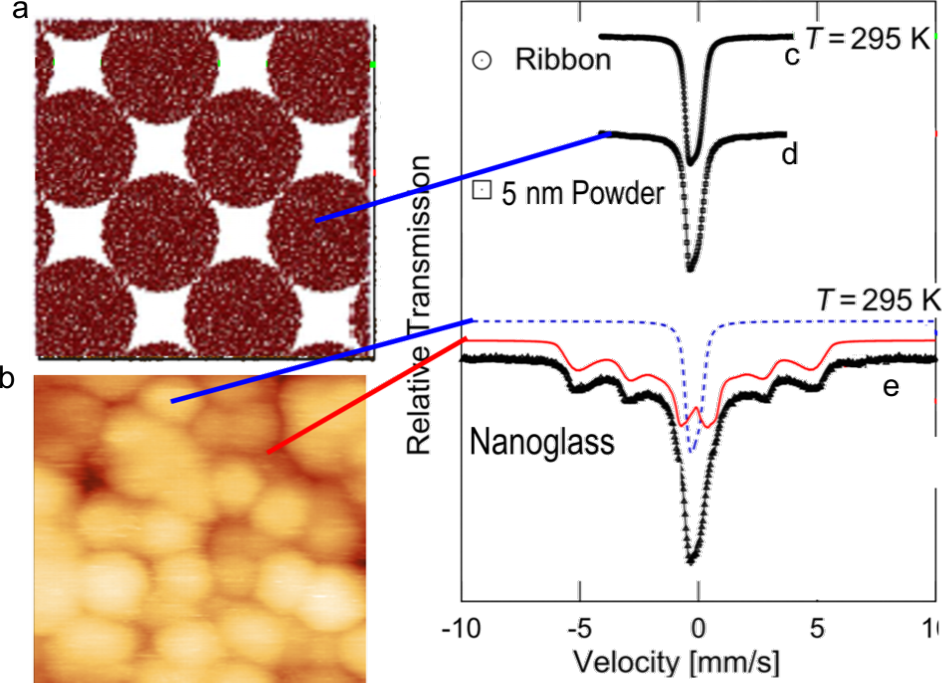
(a) As-quenched (from the vapor phase) nanoglassy grains exhibiting paramagnetic behavior, and (b) quenched from vapor phase and compacted nanoglass, in which the interface matter exhibits ferromagnetic behavior. Adapted from [15]. Copyright 2013 American Institute of Physics.

In fact even as-prepared nano-glassy powders of metallic materials do not have a core–shell structure (not more than a monolayer in any case) and exhibit a Mössbauer spectrum similar to that of the melt-cooled glasses, which indicates paramagnetism [15]. However, once the powders are cold-compacted at different pressures, a six-line spectrum characteristic of ferromagnetism is obtained, i.e., ferromagnetism originates at the interface phase in metallic nano-glasses formed by compaction (Figure 2). This view is supported by the observation that the volume fraction of the ferromagnetic material scales with the volume fraction of the interfaces [15]. This raises a question: How much lattice expansion can be tolerated in the boundary regions (compared to the grain interior) before the use of MD simulations, which consider the inter-atomic forces in the grain/interphase boundaries/interfaces to be similar to those present in the grain interior, does no longer provide valid results? The answer is not known at present.

Nano-glasses, compacted in the above fashion, are very stable (compared with their melt-quenched variants) even at temperatures close to the crystallization temperature. To date, the width of the glass–glass boundaries has not increased by a factor of more than two even after annealing the specimens close to the glass transition temperature for several hours. (Usual molecular dynamics simulations, in contrast, suggest that the glass–glass boundaries would delocalize in a few nanoseconds at 100 K.) These observations emphasize that detailed computations will be necessary in order to determine the interatomic forces in solids with modified structures/basic units and reduced densities for different materials. There are some works in which such computations based on ab initio methods, e.g., tight binding model, are described [17–18]. Similar ab initio calculations have also been undertaken for boron. It has been shown that the structural details of beta-rhombohedral boron, such as vacancies and extra occupancies, are due to the details of the electronic structure requirements and not just structural defects [19–21]. In these cases, the formation of structural/basic units will depend on the separation between the atoms and the nature (metallic, ionic or covalent) of the electronic interactions and the magnitude of the interatomic forces. The individual structural/basic units and the free volume present in them will depend on the number of atoms present in the ensemble, the start and end temperatures and other experimental conditions including the rate of heating and quenching. In view of the above complexities, an ad hoc approach, viz., the correction of existing potentials over short distances, as it is done while simulating displacement cascades under irradiation, may be attempted. But there is no guarantee that such empirical fits can be of universal relevance. In a recent experiment, even when a nano-glass was cut into very thin slices, delocalization did not take place [22]. There is a need to understand this remarkable stability.

**Figure 3:**
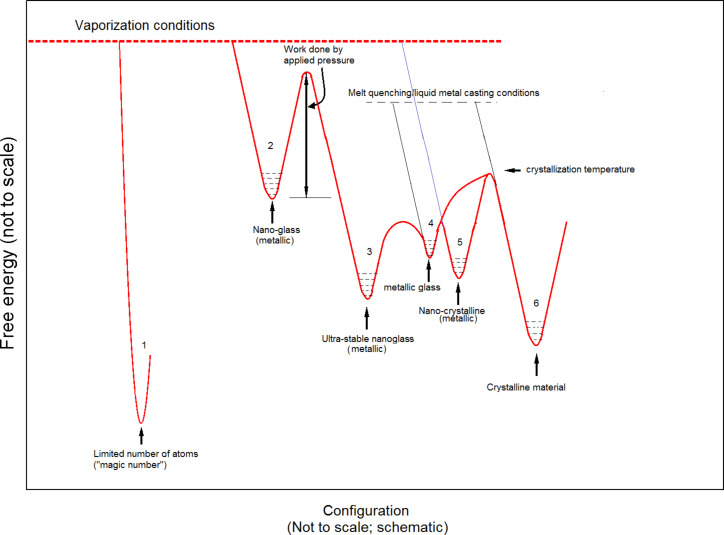
Schematic representation of phase transformations in the free energy–configuration space.

Taking the above points into account, we arrive at Figure 3, a qualitative pictorial representation in free energy–configuration space. Depending on the degree of metastability, six basic configurations are possible: (1) the maximum degree of packing and stability of the structure is obtained with a small number of atoms. We have already pointed out the limitations in the understanding of such structures at present. This configuration is different from the rest, as the others deal with a situation in which the number of atoms is above a critical value (not yet predicted). When one quenches the atoms from the vapor phase, depending on the elements present, concentration and experimental conditions, one may get (2) a nano-crystalline material, or (3) a nano-glassy material. These are metastable structures. Comparing the two, a nano-crystal appears to be more stable than a nano-glass. (In a recent experiment, annealing led to the precipitation of nano-crystals from a nano-glass [14]). The nano-glasses have properties similar to those of melt-cooled metallic glasses. The quenching is drastic and the structure is metastable. A nano-glass may also be obtained by amorphizing a nano-crystalline material, e.g., by repeated rolling and folding [23]. When such nano-glassy grains are subjected to a sufficiently high pressure (although the exact threshold value has not been established yet), the activation energy required to transform them into an “ultra-stable” nano-glassy state is provided. (It may also be possible to supply an equivalent amount of energy by heating. But this hypothesis is yet to be tested.) As noted earlier, in view of the much larger energy release (energy stored as a result of quenching from the vapor phase and triggered by subsequent application of pressure) and the consequent local temperature rise, the transformation becomes non-adiabatic. Therefore, an entropy-dominated transformation, in which the atoms are able to reach more stable configurations, comes into play. This gives rise to (4) an “ultra-stable” nano-glass. It is interesting that even when such a nano-glass is sliced very thin, its stability is intact. It is important to find out the minimum thickness of this slice or the number of atoms in the aggregate necessary to retain this enhanced stability. (5) Metallic glasses or bulk metallic glasses with basic units similar to those present in the nano-glass described in (3) (the difference is only in the free energy of the system) may be obtained directly by melt-quenching. (6) The melt-quenched glass gets converted into a nano-crystalline or crystalline material above the crystallization temperature, when the required activation energy becomes available. Alternatively, crystalline materials can also be produced directly by casting. Within each state, depending on the experimental conditions, the free energy of the system could change, i.e., each of these states of matter can be present with different free energies. Although Figure 3 is schematic and not to scale, based on empirical observations it is suggested that the relative stability of the different states of matter appears to be as indicated in the diagram. This brings one to Kauzmann’s entropy crisis [24–26], which suggests that if the entropy of many supercooled liquids is extrapolated to low temperature, the amorphous state is predicted to have a lower entropy level than the highly ordered crystal well above absolute zero. In our opinion, such a linear extrapolation may not be correct. This is because the adiabatic approximation, on which the extrapolation is based, is not valid in these cases (see above and also later).

It is emphasized that in each state depending on the experimental conditions and the number of atoms that form the structural/basic unit, the interatomic spacing and the free volume fraction could be different. When the lattice expansion is beyond a certain limit, the nature of bonds in the grain interior will be of a different kind compared with that in the interface regions. Then, differing interatomic potentials and distinctly different properties can be found in the grain interior and the interface matter respectively and this is what is seen in the case of “ultra-stable” nano-glasses. The maximum allowable lattice expansion, at which the approximation that the interatomic potential in the grain interior and at the grain/interphase boundary/interface can be considered to be similar is still valid, is yet to be determined. For a complete description of the atoms arranging themselves into structural/basic units in the interface region, a more detailed characterization of the bonds (including the differences compared with the grain interior) is required.

A number of issues need to be addressed for a quantitative understanding of the structure and properties of “ultra-stable” nano-glasses. As stated earlier, adiabatic approximation, which leads to electronic configurations of atoms in the ground and transition states being identical, may not be permissible. For example, reduced atom mobility at low temperatures may alter interatomic distances and free volume fraction. Hence the electronic configuration and the nature and magnitude of interactions could change. This is particularly important for transition metals. It is not easy to quantify the coordinated nature of movement of atoms. Moreover, the role of anharmonicity and internal stresses in determining the properties of nano-glasses is not clear. It will be quite a while before the above theoretical problems are solved rigorously. But, solutions to practical problems cannot wait for long. So approximate/semi-empirical solutions are needed.

### Limiting approximations

#### Upper limit (the simplest case)

The different ideas used to describe crystalline matter were recently examined, including situations in which long-range periodicity is missing, e.g., general high-angle grain boundaries [27]. Here it is important to note that in crystalline materials and melt-quenched metallic glasses the electronic structure, if at all different in grain/interphase boundary/interface regions compared with the grain interior, is due to an increase in defect density, as found, for example, in nano-crystalline materials produced by plastic deformation [28]. This increases only the interatomic distances in the boundary region compared with the grain interior to a limited extent, without changing the nature of the bonds. (Then, the boundaries become weaker than the grain interior because of the larger interatomic distances.) Therefore, for this class of materials molecular dynamics (MD) simulations may be used to determine the structure of grain/interphase boundaries/interfaces. (As these involve the use of empirical potentials, their relevance has to be tested first against experimental results for each case.)

The upper-limit approach treats the boundaries to be planar (an approximation). Further simplification is possible if they are classified either as tilt or twist boundaries. Then elegant treatments based on crystallographic and geometrical concepts are possible. Those who have examined grain boundaries under a microscope are aware that flat boundaries are seen very rarely and that it is not easy to classify a real boundary uniquely as a tilt or twist type. Often, one encounters mixed-types and curved surfaces. In addition, most of the experiments are on boundaries of metallic materials. These restrictions require a careful consideration whether an approach based on crystallographic concepts is applicable.

In a grain boundary of a polycrystalline material, the structure is periodic because the boundary conditions, i.e., those introduced by the crystals on both sides, are periodic. For *static* flat boundaries, the concept of coincidence site lattice (CSL) of Kronberg and Wilson [29] may be used, if the misorientation between two grains is such that it will allow certain atoms at the boundary to belong to both the crystals that form the boundary. However, if the misorientation deviates slightly from this special relationship, there will be a tendency to form low energy clusters, if necessary by adding a unit of a different structure of a low energy cluster, which belongs to another special relationship of low energy. This way, many different kinds of low energy clusters are formed to yield a given orientation relationship. In other words, the boundary structure is a 2D array of low energy clusters that form the transition layer between two crystal lattices of a given misorientation. A consequence is that low energy relationships are not necessarily CSLs. (However, some low energy grain boundary orientations may have coincident atom sites, which would qualify them as CSLs.) This has been proved in several experiments involving relaxed boundaries. For example, in rotating sphere experiments low energy relationships varied as a function of the kind of chemical bonds and temperature [30], an observation which cannot be accounted for by the purely geometrical CSL model. The latter would predict the same special boundaries for the same lattices and given misorientation. The last mentioned paper also demonstrates that there are many CSL orientations that do not result in low energy boundaries. Likewise, some cusps in the energy–misorientation plots disappear with increasing temperature, although the order in the grain boundary remains intact below the melting point. This shows that the effects of interfacial entropy are significant [31]. In addition, the average thermal expansion of grain boundaries is 2.5–5 times that of the crystalline grain interior. A purely geometrical model cannot account for such a differential change with temperature [32]. The effect of pressure is also significant. The *P*Δ*V* term causes some cusps in the energy–misorientation plot to disappear [33]. This is evidence for pressure-induced structural changes in grain boundaries and implies that grain boundaries have a larger free volume and larger interatomic distances than the grain interior and that these can be altered by compressive stresses of sufficiently high magnitude. By carrying out massive MD simulations of relaxed boundaries, Sutton and Vitek [34–36] have shown that the structural unit model, coupled to a realistic potential, results in a description, which is consistent with the current understanding of the structure of grain boundaries (the “good” crystal part) and that the predictions are in line with high-resolution TEM results concerning grain boundaries.

However, the CSL concept, which is very simple, can be used to advantage in certain situations, e.g., to explain low-angle and twin boundaries. In case of small angle boundaries the CSL description reduces to the Read–Shockley model [37] and is obtained by placing a dislocation core at regular intervals along the grain boundary. A simple atomistic model for the formation of annealing twins was proposed [38] to be the result of a 2D nucleation process on the {111} planes of growing grains of an fcc lattice. In this case, every atom at the boundary will be in a CSL position. It has already been pointed out [39] that the twin boundary is a first-order twin relationship and should be distinguished from high-angle boundaries. So the use of the CSL concept to describe “high-angle boundaries” in general [40] is perhaps not correct. However, there are boundaries beyond the low-angle range, which can be described as “sigma boundaries” [39] or “vicinal” boundaries [41], for which also the CSL concept, including the formation of a displace–shift–coincide (DSC) lattice, could be physically meaningful, with certain limits imposed on the permissible deviations from the CSL position. In this category there are four models [39,42–44], of which the one of Palumbo and Aust [39,45–46] perhaps matches experimental results (concerning boundary corrosion) more accurately. However, the Brandon criterion [42] is by far the most popular. Two more points may be noted: (a) There is little support for the general usefulness of purely geometrical models in determining grain boundary energies [41]. (b) The demonstration that for specific twinning operations (i.e., 180° rotations) along rational indices [hkl], a 3D coincidence site lattice, having Σ = h^2^ + k^2^ + l^2^ and a boundary (i.e., twinning) plane {hkl} is generated in cubic crystals [47] follows directly from Euler’s transformation rules, i.e., the mathematics is correct, but well-known.

Very recently, Raabe and coworkers [48–50] have examined the relevance of the CSL concept in understanding the two properties of segregation and corrosion by using TEM, atom probe tomography (APT) and a “pseudo” 3D-EBSD approach. The major conclusions are as follows. (a) Segregation for low-angle grain boundaries scales with the number of dislocations, i.e., it increases with an increasing boundary misorientation in the low-angle region. (b) For high-angle GBs, the coincidence number alone is not a decisive parameter, but knowledge about the GB plane is essential, i.e., whether the GB is coherent or not. For example, for Σ3 twins only fully coherent GBs with (nearly exact) (111) GB planes exhibit low segregation. (c) The occurrence of a certain coincidence lattice is not a sufficient criterion for a GB to be “special”. This is because the coincidence site lattice defines only three (out of the five) degrees of freedom of a GB and it does not provide any information about the orientation of the GB plane and the degree of coherency in it. For instance, the Σ9 GB, in spite of being a low ΣCSL boundary, does not exhibit “special” behavior regarding its resistance to segregation. This is attributed to the fact that the studied Σ9 boundary is not symmetric, i.e., the Miller indices of the boundary plane measured with respect to the two mutual grains are not identical, and therefore, also do not correspond to a coherency plane. (d) In case of corrosion, only the low-angle boundaries and the Σ3 and Σ5 boundaries do not get attacked. Other low-angle boundaries get attacked to a low extent. But other low ΣCSL boundaries, Σ3 ((81d) (ideally ≈60.4° <443>; but ≈60.1° <443> in the experiment; a fourth order twin which qualifies as Σ3 according to the Brandon criterion)), Σ27 boundaries (a third-order twin boundary, often formed in materials with low stacking fault energies), and random high-angle GBs get attacked severely. A Σ9 boundary is attacked to a moderate to high degree. Sometimes even some random high-angle grain boundaries do not get attacked. All the above calculations regarding the boundaries are based on the deviations allowed by the Brandon criterion, which seems to be too “generous”. Thus the usefulness of the CSL concept in relating the structure to the properties beyond low-angle and coherent boundaries is not clear.

Notwithstanding the above statements, it is safe to say that the crystallographic approach has helped to achieve a remarkable simplification of a problem, as long as the crystals contain only a small number of defects. Then, idealization in terms of planar boundaries, for which crystallographic notions make perfect sense, is possible. The defects are taken into account by breaking the symmetry rules using well-calibrated local considerations, again derived from geometrical notions. But, when the defects are numerous and the boundaries are far from being linear, e.g., a general high-angle grain boundary in which long-range periodicity is also missing, certain parts of the boundary contain significant free volume and describing them in crystallographic terms becomes difficult. The approach breaks down and an alternative description based on the nature and magnitude of interatomic forces becomes essential. It must be mentioned that for the latter approach, which is more realistic/rigorous, so far only the method (MD simulations) of working out the structure of grain boundaries by assuming suitable empirical interatomic potentials for simple materials has been spelled out. The procedures for extending this technique to include alloys of commercial significance are yet to be developed [27]. Enormous efforts that will involve ab initio methods and take into account interatomic (including electronic) interactions will be necessary to improve the situation.

#### Lower limit: the concept of representative volume (with a focus on the region of disorder)

**Figure 4:**
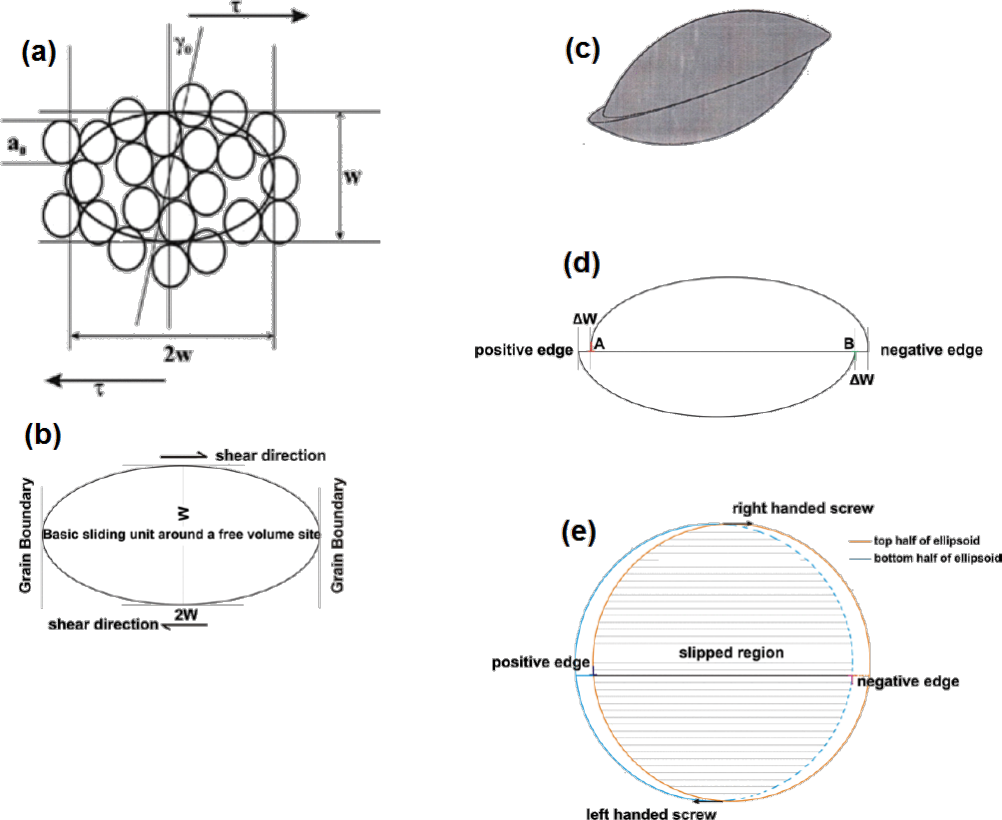
(a) Sliding/Shear unit, according to the GBS Model. (b) Elevation view of the undeformed oblate spheroid. (c) Isometric view of the deformed oblate spheroid. (d) Elevation view of the deformed oblate spheroid. (e) Plan view of the deformed oblate spheroid. Reproduced with permission from [27]. Copyright 2012 Elsevier.

**Figure 5:**
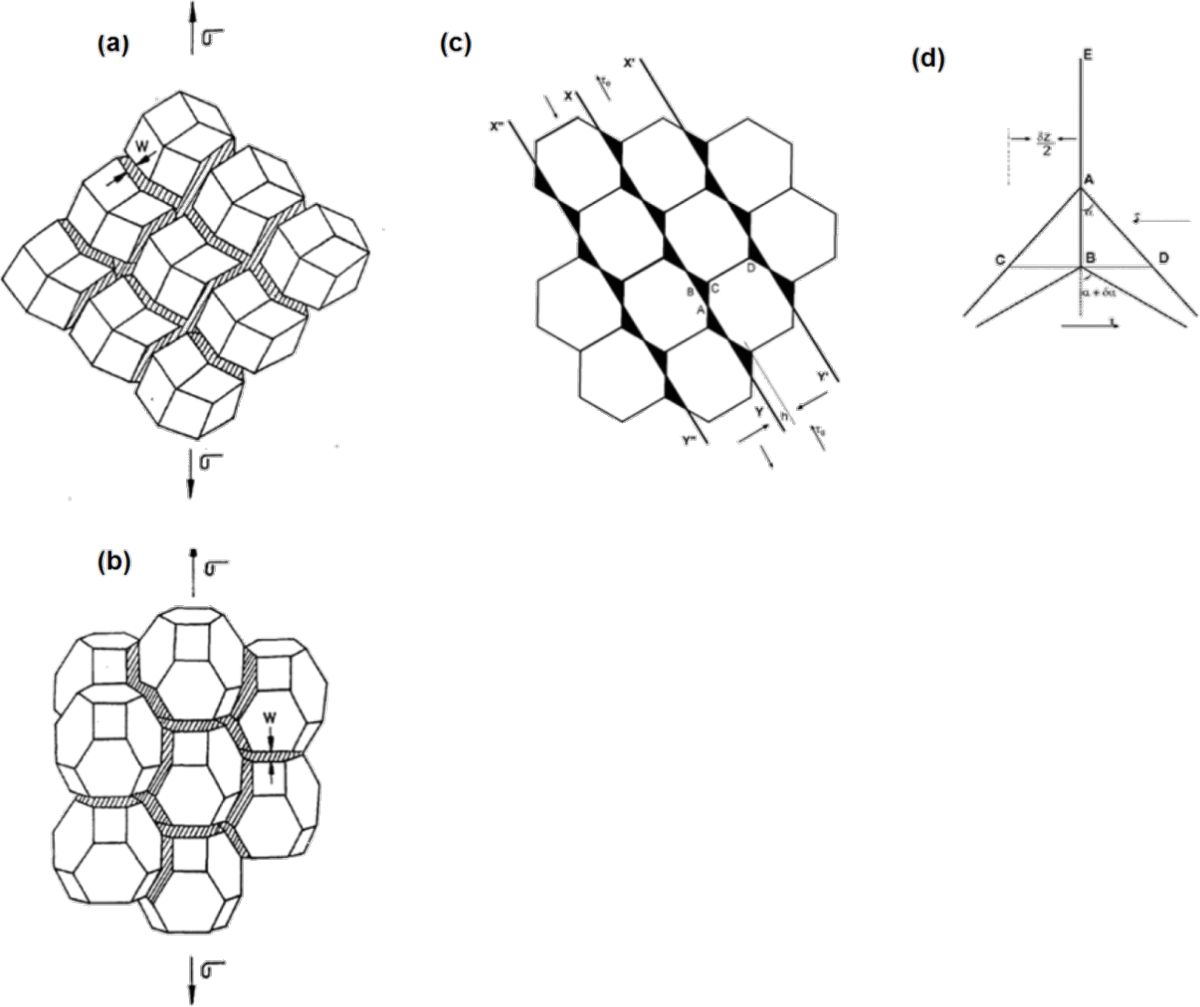
Development of mesoscopic grain/interphase boundary sliding. Shaded grain boundaries of rhombic dodecahedral (a) and tetrakai decahedral (b) grains within which the rate-determining process is confined. (c) Resulting planar interfaces (along XY, X’Y’, X”Y”, etc.), which are 2D sections of an aggregate of grains of equal size and rhombic dodecahedral shape. When the atoms located in the shaded regions are moved by the extension of the boundaries normal to the shear direction to reach the sliding boundary planes, a plane interface results. (d) Shear-stress driven movement of a boundary triple junction to lower the overall free energy of the system. (a) and (b) reproduced with permission from [54], Copyright 1996 Maney Publishing and (c) and (d) reproduced with permission from [56], Copyright 2004 Elsevier.

It was pointed out [27] that a geometry-based approach does not seem to lead to useful results in case of general high-angle grain boundaries, in which long-range periodicity is missing and significant free volume is present at certain misorientation-dependent locations in whose vicinity the structure cannot be described in crystallographic terms. But the high-angle boundary is the most important structural element needed for describing superplastic flow. Interestingly, superplasticity is present in both crystalline and glassy materials [51–53]. Postulating: (a) that the rate-determining process during superplastic deformation is confined to basic units (not crystalline) around free volume sites in the high-angle grain/interphase boundary/interface, and (b) that the sliding along grain/interphase interface/boundaries is the rate-determining process, steady state superplastic deformation has been explained quantitatively by using the concept of “representative volume”, often invoked in analyses of transport phenomena in large systems (involving heat, mass and momentum transfer). The representative volume is assumed to be large enough to allow the operation of the unit processes relevant to a problem on hand. Simultaneous operation of these unit processes in different representative volumes that are present throughout the system and the summation of their effects is assumed to result in the overall response. Such an approach presupposes that the real problem is so complex that a rigorous analysis in all aspects is not possible. (The assumption of “representative volume” is used even when the upper limiting case, based on the assumption that grain boundaries are flat, is involved, e.g., Nabarro–Herring (N–H) creep, even though this is not stated explicitly. In N–H creep, for example, it is assumed that what occurs in a cubic grain is repeated all over the sample.) For a quantitative understanding of steady state superplastic flow [53–57], the basic unit of boundary/interface sliding, the representative volume, is assumed to be an oblate spheroid of about five atomic diameters along the boundary plane and about two and a half atomic diameters (average grain boundary width [58]) in the perpendicular direction. (A deformation of oblate spheroids of such dimensions along the interface between glassy regions in case of metallic glasses would lead to the formation of shear transformation zones, described by Argon [59] and others.) As mentioned above, the basic units of boundary/interface sliding are defined around free volume sites, which are present at discrete places characteristic of the interatomic forces and the boundary misorientation. The sequential shear of such oblate spheroids, when it reaches the end of a boundary, results in a boundary-sliding offset and, in contrast, deformation would be blocked, if a triple junction were encountered. For continued boundary sliding, this sliding process has to develop to a mesoscopic scale through the formation of plane interfaces. The driving force for the formation of such plane interfaces is the minimization of the free energy of the system and the fact that for this configuration, the applied stress does maximum work (principle of maximum work of G. I. Taylor). These processes are illustrated in Figure 4 and Figure 5. Depending upon the boundary misorientation, which, along with the nature and magnitude of the interatomic forces, decides the free volume present inside an oblate spheroid and free volume fraction along a boundary, the neighborhood of the deforming oblate spheroid would be different. As a result, the internal stress distribution that develops due to sliding will change with material chemistry, boundary misorientation, stress, and strain. A way of quantifying the internal stress distribution is then spelled out [54]. It is pointed out here that the effects of composition, atomic arrangement in the oblate spheroid, impurity/solute/dopant effects and free volume present are captured into two phenomenological parameters, the free energy of activation, Δ*F*_0_, and the threshold stress necessary for the onset of mesoscopic boundary/interface sliding, τ_0_. The above ideas have been shown to be useful in understanding quantitatively superplastic deformation in micro-grained and nanostructured pseudo-single phase as well as micro-duplex metallic materials, ceramics, ceramic composites, intermetallics, dispersion strengthened alloys and an alloy containing quasi-crystalline particles [53]. Very recently, it has been found to be useful for interpreting superplastic flow in bulk metallic glasses also (Buenz, J.; Padmanabhan, K. A.; Wilde, G., unpublished work). Experimental support for mesoscopic boundary sliding/plane interface formation and near-random grain rotation due to the rate-determining boundary/interface sliding process is also available. These are summarized in [53–57,60–62].

The shear modulus and the free volume present in the basic sliding unit, γ_0_, (composition, impurity/solute/dopant content dependent) can be determined by using ab initio calculations, in particular the tight binding model, which is computationally less intensive. Such a computation will, however, result only in values for the above parameters for the atom cluster of oblate spheroid shape. But, in the real situation this cluster is embedded in a solid matrix. Eshelby [8] has already shown how such an insertion process modifies the important engineering properties of the atom cluster (treating it as an inclusion). In turn, by using these values and the equations (3), (5) and (6) of [27] the values of Δ*F*_0_ and τ_0_ can be computed. These topics dealing with ab initio calculations form the subject of a present investigation.

It must be noted that an attempt to describe the numerous general high-angle boundaries of differing misorientations in terms of a unique representative volume, an oblate spheroid, was made to develop a deterministic mathematical model. In reality, the basic units of sliding are numerous and are likely to have different shapes (even irregular), numbers of atoms and free volume fractions. Then, there will be a spectrum of values for Δ*F*_0_ and τ_0_. This will lead to a stochastic model, with two additional empirical values, the mean and the standard deviation for the above two parameters. Such a model may be open to criticism that it is a curve-fitting exercise. Therefore, a stochastic model was not developed. However, it is important to note that for understanding other properties, the structural element of interest may have to be represented as a combination of more than one “representative volume”.

This is a continuum model. But, an examination of Figure 4 reveals that the deformation of oblate spheroids proceeds along a grain boundary leaving behind circular loops. By definition, they constitute dislocation loops of zero Burgers vector in the Volterra sense. (They cannot, however, be described in terms of crystallographic concepts because the loop formation displaces the basic sliding unit in the direction of stress only by about a tenth of the interatomic spacing in the boundary region.) In this sense, the speculation found in the literature that the motion of extrinsic boundary dislocations could cause grain boundary/interface sliding is justified. Assuming the extrinsic boundary dislocations to be discrete, Nazarov and coworkers (for a summary see [63]) have been able to predict accurately the grain boundary excess energy and the additional free volume/lateral expansion found in severely plastically deformed materials. Now Nazarov and Valiev (personal communication) believe that their model is “mesoscopic” and thus the need to explain the extrinsic boundary dislocations in terms of a crystallographic notion is dispensed with. By using MD simulations, an investigation has just been started to verify whether the propagation of “dislocation loops” in our continuum approach is equivalent to the propagation of extrinsic boundary dislocations assumed for boundary sliding in the analysis of Nazarov et al. [63].

#### More complicated situations

So far two extreme cases were examined in detail. (a) Considering the entire boundary to be planar and understanding the responses of the boundaries to external stimuli by using notions based in crystallography. This covers all kinds of crystalline grain boundaries, excluding random high-angle grain boundaries. Here two alternative approaches are found: (i) a purely geometrical approach based on coincidence site lattice concept, and (ii) the structural unit model, which emphasizes that structures (including grain boundaries/interfaces) develop because of the presence of interatomic (including electronic) interactions/forces and, for a given set of experimental conditions, because of a competition between the minimization of free energy and the maximization of entropy of the system. A survey of contemporary literature reveals that the latter approach is the favored one. However, in some situations, the former (geometrical) approach could help solve problems easily, albeit approximately.

In the *lower bound approximation*, by assuming that all steps involved in the rate-determining process are found inside a *basic unit of atoms in the boundary* of atomistic dimensions, and where the structure is not crystalline and more open than in the grain interior and the rest of the boundary, a mathematical treatment has been developed for the high-angle boundary dominated process of superplasticity, which is observed in both crystalline and glassy materials. These basic units are present at various places in the boundary between (crystalline) structural units, with their (basic units) locations being decided essentially by the boundary misorientation and the nature and strength of the interatomic forces. The shape, size and properties of the basic unit is the result of a complex interaction between interatomic (including electronic) forces, free energy minimization and entropy maximization [51–57]. When no assumptions are made about the shape and size of the basic unit, the number of atoms present in it and the enclosed free volume fraction also are variables. For *mathematical* development, the shape of the basic unit was *approximated to* an oblate spheroid of 5 atomic diameters in length and 2.5 atomic diameters in height. This results in a deterministic model that estimates the overall (average) response of the system in the steady state to an external stress with regard to some useful properties. Needless to say, for studying local and topological effects, the shape, size and free volume fraction of every basic unit in the boundary should be considered, along with their neighborhoods. This is a formidable problem and is yet to be solved.

It is seen experimentally that in many cases, in addition to short-range order, intermediate order as well as long range order and periodicity/quasi-periodicity (in the latter case, crystalline/quasi-crystalline matter) are present. Then, it will be possible to start with a “representative volume” of larger dimensions, with accompanying modifications/simplifications. For the simplest case of the entire boundary being regarded as a plane, this has been done by using elegant geometrical/crystallographic notions.

The “ultra-stable” nanoglasses, produced by quenching from the vapor phase and the powders subsequently compacted, seem to be in a category of their own, whose quantitative description would require the conversion of the free volume fraction in the oblate spheroids of the lower limit to a widely varying variable (with far more free volume fraction than what is needed to explain the structure of random high-angle boundaries and melt-quenched metallic glasses) so much so that when the free volume fraction exceeds a critical value (yet to be calculated) the very nature of the chemical bonds between the atoms for the same chemical composition changes, e.g., from metallic to covalent. In boundaries of this type of nano-glasses, such highly expanded basic units coexist with (crystalline) structural units (responsible for the unique type of short-range order seen), which could fit into the rest of the boundary by a process similar to what is envisaged in the “lock-in” model [64–65], and the type of basic units considered while discussing high-angle grain boundaries/melt-quenched metallic glasses/superplasticity. Melt-quenched metallic glasses and nano-glassy powders (not compacted), in contrast, are not likely to have the highly expanded basic units in which the nature of the chemical bonds has changed. In this sense, melt-quenched metallic glasses and nano-glassy powders are similar to a relaxed high-angle boundary, with only a difference in the free volume fraction, the proportion of structural and basic units, the spatial distribution of these units and the free volume. The experimental observation (S. V. Divinski, personal communication) that diffusivity in melt-quenched metallic glass is between those of volume diffusion and grain boundary diffusion in crystalline materials, all of which are less than that of severely plastically deformed grain boundaries, seems to be in line with this idea. Construction of 3D structures that incorporate these ideas is likely to be a challenge.

There is also a difference in the boundary between two solitons (crystals), which is an inter-crystalline/interphase boundary, and the boundary between two glassy regions (grains) that are not solitons. If one examines the distribution width of the hyperfine field of the glass component and the interfacial component in Figure 2, one finds that they are comparable. Both phases (glassy component and the interfacial component) fluctuate structurally within a specimen by the same amount. In contrast, in a poly-crystalline or nano-grained material, the component of the spectrum associated with the crystallites is very narrow, while the one associated with the boundaries is so broad that it appears almost like a uniform background, i.e., there are wide fluctuations in the structures of the boundaries. This difference is traced to the fact that a glass has shear modulus that tends to zero as time tends to infinity. In case of the interfacial phase, if there is a minimum in the free energy–free volume space at a different free volume fraction than in the glassy component, all the boundaries in the nano-glass will, in the long run, work their way into that minimum. Evidently, such a process is not possible in inter-crystalline boundaries because they will have a “structural memory” due to the given misorientation between the crystals, which have time-independent shear moduli. For further consideration, the reader is referred to [22].

Finally, there is the question why long-range order and periodicity/quasi-periodicity should develop at all in materials. We see the following possibilities for a mathematical development. (a) The total free energy of the system becomes less than what is the case in their absence (the traditional approach). (b) The total free energy of the system, which is a function of several variables, experiences a bifurcation at some point. If this function has a value more or less than a certain figure (which will depend on the way the function is defined), it will have long-range periodicity/quasi-periodicity and be crystalline/quasi-crystalline. Otherwise, the structure would be non-crystalline. (c) The fractal nature of microstructures has been well documented [2–3], including that present in a nano-glass [14]. Is it possible that the development of long-range periodicity/quasi-periodicity and crystalline/quasi-crystalline structure corresponds to the “strange attractors” of the Chaos theory?

## Conclusion

It is concluded that a generalized structural/basic unit (crystalline, non-crystalline or of any scale) model, which takes into account the interatomic (including interelectronic) interactions, the spatial distribution of atoms and electrons, the number of atoms and free volume fraction in the ensemble, the starting and finishing temperatures, rate of heating/cooling and other experimental conditions can describe in a general manner the structures that develop in grain and interface boundaries in crystalline/quasi-crystalline and glassy materials. As a quantitative development that takes all these variables into account is rather difficult, we have arranged them in increasing order of complexity. By assuming the boundaries to be flat/planar, simplified solutions can be obtained by using crystallographic notions. At the other extreme, by using the concepts of short-range order and “representative volume”, which assumes that a relatively much smaller volume taken from the whole is large enough to simulate all relevant steps connected with a rate-determining process, simplification is achieved to explain a high-angle boundary dominated process like superplasticity, found in crystalline as well as melt-quenched (bulk) metallic glasses. The method for a quantitative description of more complex situations, where (crystalline) structural units, basic units (which include some free volume and crystallinity is absent, as found in high-angle grain boundaries and melt-quenched metallic glasses) and non-crystalline regions of far greater free volume fraction (where the nature of interatomic (including electronic) interactions could be different from that in the grain interior) which coexist (as found in compacted nano-glasses), is yet to be developed.
